# Rheumatoid arthritis bone marrow environment supports Th17 response

**DOI:** 10.1186/s13075-017-1483-x

**Published:** 2017-12-08

**Authors:** Ewa Kuca-Warnawin, Weronika Kurowska, Monika Prochorec-Sobieszek, Anna Radzikowska, Tomasz Burakowski, Urszula Skalska, Magdalena Massalska, Magdalena Plebańczyk, Barbara Małdyk-Nowakowska, Iwona Słowińska, Robert Gasik, Włodzimierz Maśliński

**Affiliations:** 1 Department of Pathophysiology and Immunology, National Institute of Geriatrics, Rheumatology and Rehabilitation (NIGRR), Spartanska 1, 02-637 Warsaw, Poland; 2 Department of Pathology, National Institute of Geriatrics, Rheumatology, and Rehabilitation (NIGRR), Warsaw, Poland; 3 Department of Diagnostic Hematology, Institute of Hematology and Transfusion Medicine, Warsaw, Poland; 4 Department of Rheumoorthopaedic Surgery, National Institute of Geriatrics, Rheumatology and Rehabilitation (NIGRR), Warsaw, Poland

**Keywords:** Bone marrow, IL-17, IL-15, CCL20, Rheumatoid arthritis

## Abstract

**Background:**

Rheumatoid arthritis (RA) is a systemic, autoimmune disease leading to joint destruction and ultimately disability. Bone marrow (BM) is an important compartment in RA, where pathological processes from “outside the joint” can occur. IL-17 is a cytokine that exerts proinflammatory effects and participates in the process of bone destruction. It is believed that IL-17 is involved in pathogenesis of RA. However, little is known about the biology of this cytokine in BM. In the present study we investigated Th17-related cytokines in RA BM.

**Methods:**

BM samples were obtained from RA and osteoarthritis (OA) patients during total hip replacement surgery. Levels of IL-17AF, IL-17AA, IL-17FF, IL-1β, IL-6, IL-23, TGF-β and CCL20 in BM plasma were determined by specific enzyme-linked immunosorbent assay tests. Percentage of IL-17-producing cells in BM was evaluated by flow cytometry. The effect of IL-15 stimulation on IL-17 production by BM mononuclear cells was examined in vitro.

**Results:**

Increased levels of IL-17AF were observed in BM plasma of RA patients in comparison to OA patients. Increased concentrations of IL-1β, IL-6 and CCL20 were observed in RA compared to OA BM plasma. Concordant with these findings, significantly increased percentages of CD3^+^CD4^+^IL-17^+^ and CD3^+^CD4^+^IL-17^+^IFN-γ^+^ cells were present in RA BM in comparison to OA BM samples. Finally, abundant in RA BM, IL-15 increased IL-17 production by cultured BM mononuclear cells.

**Conclusions:**

In the course of RA, the BM microenvironment can promote the development of Th17 cell responses and overproduction of IL-17AF that may lead to increased inflammation and tissue destruction in RA BM.

## Background

Rheumatoid arthritis (RA) is a systemic, autoimmune disease leading to joint destruction and ultimately disability [1]. Data obtained in the last decade indicate that bone marrow (BM) is an important compartment in RA, where pathological processes from “outside the joint” can occur. The cellular infiltrates found in RA BM consist of immunological cells that may form aggregates resembling germinal centres in secondary lymphoid organs [2]. Our previous flow cytometry analyses showed an increased number of mononuclear cells and accumulation of activated T cells and B cells in the BM of RA patients in comparison to osteoarthritis (OA) patients [2, 3]. Increased levels of proinflammatory cytokines and chemokines in RA BM were also observed, indicating an ongoing inflammatory process in this compartment. One of these cytokines is IL-15, which can be involved in T-cell activation [2, 4–6].

Interleukin-17 is predominantly expressed by a specific subset of human T-helper cells—Th17 cells. In addition, recent evidence indicates that IL-17 could also be produced by several innate immune cells and activated or inflammatory T cells [7]. It is assumed that this cytokine overproduction plays a crucial role in inflammation and the development of several autoimmune diseases, including RA. However, little is known about biology of this cytokine in BM. There are six known isoforms of IL-17 (IL-17A–IL-17 F). Th17 cells are able to produce only proinflammatory IL-17A and IL-17 F, where IL-17A is considered more potent than IL-17 F. These two isoforms create dimers: in body fluids, homodimers IL-17AA and IL-17FF and also heterodimer IL-17AF could be detected [8]. Differentiation of human naïve T cells into Th17 cells requires the presence of TGF-β and at least one of the following proinflammatory cytokines: IL-1β, IL-6, IL-21 and IL-23 [9, 10]. In turn, Th1-related cytokine IFN-γ and Th2-related IL-4 suppress differentiation of Th17 cells [11, 12]. The proinflammatory cytokine IL-15 may also be involved in IL-17 production. Concentrations of IL-17 were demonstrated previously to correlate with concentrations of IL-15 in both serum and synovial fluid of RA patients [13]. Macrophage inflammatory protein-3α (MIP-3α/CCL20) has been reported to preferentially attract Th17 cells (via CCR6 binding) to the inflamed rheumatoid joints and the small intestine [14, 15].

In the present study we hypothesized that the RA BM microenvironment supports the development of Th17 cells and Th17 cell response. To verify this hypothesis, we examined the frequency of Th1, Th2 and Th17 cell populations as well as the concentrations of cytokines involved in Th17 cell differentiation and migration in BM from RA and OA patients. We analysed the concentrations of IL-17AA, IL-17AF and IL-17FF in the BM plasma and peripheral blood plasma of RA and OA patients. Moreover, because of the potential stimulatory effect of IL-15, the impact of this cytokine on IL-17 production has also been evaluated in vitro.

## Methods

### Patients

BM and peripheral blood were obtained from patients with RA and from patients with OA diagnosed according to American College of Rheumatology revised criteria for RA or for OA [16, 17]. Peripheral blood and BM samples were obtained from patients undergoing total hip replacement surgery. Patients’ demographic and clinical characteristics are summarized in Table 1.

**Table 1:** Patients’ demographic and clinical characteristics

	RA (*n* = 67)	OA (*n* = 43)
Age, median (minimum–maximum)	58 (31–69)	59 (30–69)
Sex, female/male	54/15	42/21
ESR (mm/h), median (minimum–maximum)	37 (5–91)	11 (2–38)
CRP (mg/l), median (minimum–maximum)	21 (0–74)	3 (0–28)
Methotrexate	40	0
Steroids	54	0
Biologics	0	0

*CRP* C-reactive protein, *ESR* erythrocyte sedimentation rate, *OA* osteoarthritis, *RA* rheumatoid arthritis

### Immunohistochemistry

Bone marrow samples obtained from six OA patients and six RA patients were examined histopathologically. BM samples were fixed in Oxford fixative (formaldehyde 40%, glacial acetic acid 2%, sodium chloride 8.7%, distilled water), routinely processed and embedded in paraffin wax. Sections 3 μm thick were cut and stained with haematoxylin and eosin. The following antibodies were then used for further staining: anti-CD8 (polyclonal Ab, dilution 1:50; Dako, Glostrup, Denmark), anti-CD4 (clone 4B12, dilution 1:10; Novocastra, now part of Leica Microsystems, Wetzlar, Germany) and anti-IL-17A (dilution 1:50; Santa Cruz). Staining was performed according to the manufacturer’s instructions. The EnVision Detection System (Dako Denmark A/S, Glostrup, Denmark) was used for detection. Positive controls were performed on human tonsils. Negative (isotype) controls were performed using ready-to-use FLEX Negative Control Mouse (cocktail of murine IgG1, IgG2a, IgG2b, IgG3 and IgM, code number IR750; Dako Denmark A/S). Samples were reviewed for expression of these proteins by a qualified histopathologist who was blinded to outcome. Appropriate cellular localization for immunostaining was membrane for CD8 and CD4 and cytoplasmatic for IL-17A. All photographs were taken using Olympus microscope cameras: DP72 Olympus BX63 and DP12 Olympus BX (Olympus, Tokyo, Japan).

### BM plasma concentration of cytokines

Bone marrow plasma samples were obtained as we described previously [2, 4]. Concentrations of tested cytokines IL-17AA, IL-17AF, IL-17FF, IL-17A, TGF-β, IL-23, IL-1β, TNF-α, IL-4, IFN-γ, IL-6, CCL20 and IL-15 were detected using specific enzyme-linked immunosorbent assays (ELISAs). All measurements were performed in duplicate.

Concentrations of homodimer IL-17AA, homodimer IL-17FF and heterodimer IL-17AF were analysed by respective ELISA Ready-SET-Go kits (eBioscience, San Diego, CA, USA) according to the manufacturer’s instructions. The detection limits were 4 pg/ml for IL-17AA, 16 pg/ml for IL-17FF and 30 pg/ml for IL-17AF.

Concentrations of TGF-β, IL-15, IL-1β and CCL20 were measured by respective ELISA Duo Set test (R&D Systems, Minneapolis, MN, USA) according to the manufacturer’s instructions. The detection limits were 31 pg/ml for TGB-β, 3.9 pg/ml for IL-1β and 15.6 pg/ml for CCL20 and IL-15.

The concentration of IL-23 was assessed using polyclonal rat IgG anti-IL-23p19 as a coating Ab and biotinylated mouse IgG anti-IL-23 p40/70 as a detecting Ab (both Abs from Nautec, eBiosciences, San Diego, CA, USA). After staining with detecting antibody, samples were incubated with streptavidin conjugated with horseradish peroxidase (Sigma). Recombinant human IL-23 (R&D Systems) was used as a standard. The peroxidase reaction was developed using *o*-phenylenediamine dihydrochloride (Sigma). The optical density was measured at 492 nm with an automatic ELISA reader. The detection limit was 15 pg/ml.

Concentrations of IL-6 were analysed as described previously [18].

### Isolation, culture and IL-15 stimulation of BM mononuclear cells

Bone marrow mononuclear cells (BMMC) were isolated by density gradient centrifugation using Ficoll-Paque (GE Healthcare Bio-Sciences, Uppsala, Sweden). Cells (2 × 10^6^/ml) were cultured in 24-well plates (Nunc, Roskilde, Denmark) in RPMI 1640 medium (Invitrogen, Paisley, UK) supplemented with 2 mM l-glutamine (Invitrogen), 10% heat-inactivated fetal calf serum (FCS) (Biochrom AG, Berlin, Germany), 100 U/ml penicillin, 100 μg/ml streptomycin (both antibiotics from Polfa Tarchomin, Warsaw, Poland), 30 μg/ml kanamycin (Sigma, St Louis, MO, USA) and 1 mM HEPES (Invitrogen) for 24 hours. For IL-15 stimulation, BMMC were cultured for an additional 72 hours in the presence of IL-15 (25 ng/ml) (R&D Systems).

### Flow cytometry evaluation of IL-15 receptor complex expression on CD3^+^CD4^+^ cells

To estimate the surface expression of IL-15R receptor complex (CD122, CD132, CD215), BMMC were washed first in PBS (without Mg^2+^/Ca^2+^) buffer containing 1% BSA and 0.06% NaN_3_, and then with glycine buffer (0.1 M, pH 3.0). In the next step BMMC were stained with antibodies anti-CD3-APC-Cy7, anti-CD4-PeCy7 (Becton Dickinson, San Diego, CA, USA), anti-CD122-FITC, anti-CD132-APC and anti-CD215-PE (R&D Systems). After the washing step, cells were incubated with 7-AAD to stain and exclude dead cells. Cells were acquired and analysed using FACSAria and Diva software (BD).

### Measurement of secretory and intracellular IL-17 production upon IL-15 stimulation

Supernatants from BMMC cultured with or without IL-15 were collected for secretory IL-17A concentration measurement using Quantikine ELISA (R&D Systems) according to the manufacturer’s instructions. Supernatants from unstimulated cell cultures served as control. In experiments designed for intracellular staining, BMMC were treated with PMA, ionomycin and GolgiStop 6 hours before the end of the culture. After staining of respective membrane antigens using anti-CD3 APC, anti-CD4 FITC and anti-CD8 APC-Cy7 Abs, cells were fixed and permeabilized. The cells were then stained for intracellular expression of IL-17A using anti-IL-17A Ab conjugated with PE. Washed cells were acquired and analysed using FACSAria and Diva software (BD).

### Flow cytometry evaluation of Th1/Th2/Th17 cell subsets in BM

Freshly isolated BMMC from patients’ BM samples were treated for 6 hours with PMA (50 ng/ml) and ionomycin (1 μg/ml) in the presence of GolgiStop protein transport inhibitor. The cells were then harvested and stained for respective membrane antigens using anti-CD3 PerCp-Cy5.5, anti-CD4 Pe-Cy7 and anti-CD8 APC-Cy7 murine Abs. In the next step, cells were fixed and permeabilized using BD Cytofix/Cytoperm kit. Subsequently, intracellular staining using anti-IL-17A-PE, anti-IFN-γ-FITC and IL-4-APC Abs were performed. After the washing step, cells were acquired and analysed using a FACSAria cell sorter/cytometer and Diva software. All used reagents were purchased from Becton Dickinson (San Jose, CA, USA).

### Statistical analysis

Data were analysed using GraphPad Prism 6 software. As obtained data were not normally distributed, according to the D’Agostino–Pearson omnibus normality test and the Shapiro–Wilk normality test, non-parametric tests were used for estimation of statistical significance of results. Comparisons between RA and OA were analysed by two-tailed Mann–Whitney *U* test. Correlations between the concentrations of cytokines were assessed using the Spearman test. The differences in IL-17 production after IL-15 stimulation were tested for statistical significance using the Wilcoxon test. *p* < 0.05 was considered statistically significant. Data are shown in the text as the median. Data are shown in the figures as the median with interquartile range or dots representing individual results.

## Results

### IL-17-positive cells are present in BM of RA patients

**Fig. 1:**
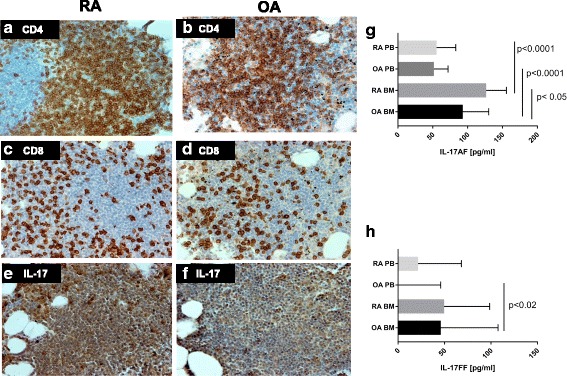
**a–f** Immunohistochemical staining of BM samples obtained from patients with RA (*n* = 6) and OA (*n* = 6); EnVision stain, ×200. **a, b** CD4^+^ T cells in lymphoid follicle. **c, d** CD8^+^ T cells in lymphoid follicle. **e, f** IL-17A expression in lymphocytes in lymphoid follicle. **g** Concentration of IL-17AF in OA and RA BM plasma (OA *n* = 32; RA *n* = 28) and in OA and RA blood plasma (OA *n* = 21; RA *n* = 15). **h** Concentration of IL-17FF in OA and RA BM plasma (OA *n* = 34; RA *n* = 28) and in OA and RA blood plasma (OA *n* = 23; RA *n* = 15). Differences between groups were calculated using Mann–Whitney *U* test. BM bone marrow, IL interleukin, OA osteoarthritis, PB peripheral blood, RA rheumatoid arthritis

Immunohistopathological examination showed the presence of IL-17A-positive cells in BM samples obtained both from RA and OA patients (Fig. 1a–f). In immunohistochemical experiments we used an antibody against IL-17A monomer. We consider that positive staining with this antibody reflects the presence of both IL-17AA and IL-17AF dimers. Importantly, using ELISA, we found statistically significantly higher concentration of IL-17AF heterodimer in BM plasma of patients with RA (126.6 pg/ml, *n* = 28) compared to OA (92.96 pg/ml, *n* = 32) (Fig. 1g), indicating enhanced secretion of IL-17AF in RA BM. It is noteworthy that in both groups of patients the concentration of IL-17AF heterodimer was higher in BM plasma than in peripheral blood plasma. Surprisingly, there were no differences in IL-17FF homodimer concentration between OA BM (45.98 pg/ml) and RA BM (49.59 pg/ml) (Fig. 1h). The IL-17AA homodimer was detected only in 3 of 32 OA samples and 2 of 28 RA samples (data not shown). These results support other authors’ observations that IL-17A exists mainly as an IL-17AF heterodimer [19]. Since on the basis of our experiments IL-17AA is hardly detectable in RA and OA BM plasma and IL-17FF levels are similar in both groups of patients, in the next flow cytometry experiments only intracellular expression of IL-17A was studied and positively stained cells were considered as the cells producing IL-17AF heterodimer.

### Frequency of IL-17-positive cells is increased in RA BM

**Fig. 2:**
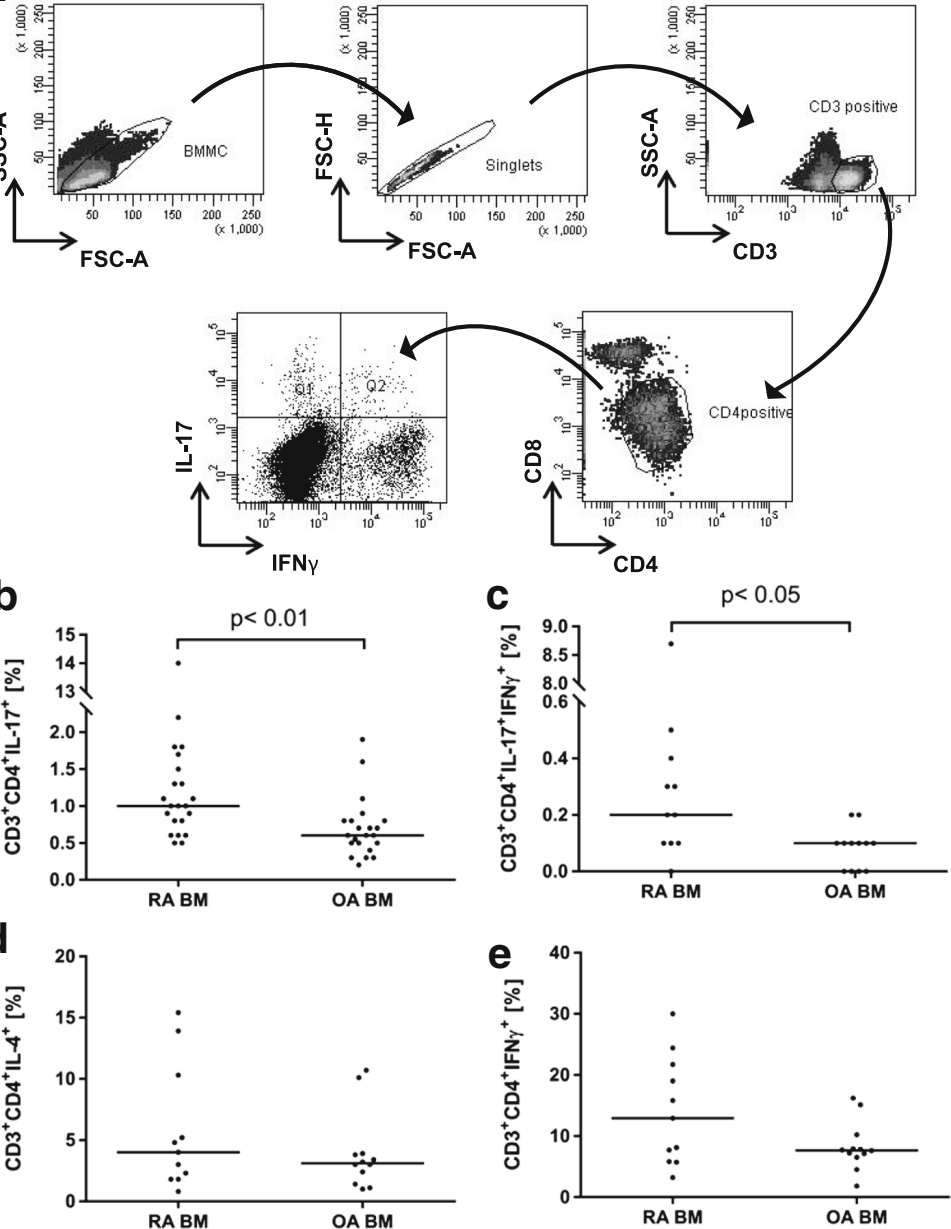
Flow cytometry analysis of Th1, Th2 and Th17 lymphocyte subpopulations in OA and RA BM. **a** Representative gating strategy for FACS of RA BMMC, showing CD3^+^CD4^+^ lymphocytes with intracellular staining for IL-17 and IFN-γ after PMA/ionomycin stimulation. **b** Percentage of CD3^+^CD4^+^IL-17A^+^ cells (OA *n* = 22; RA *n* = 22) **c** Percentage of CD3^+^CD4^+^IL-17A^+^IFN-γ^+^ cells (OA *n* = 12; RA *n* = 11). **d** Percentage of CD3^+^CD4^+^IL-4^+^ cells (OA *n* = 12; RA *n* = 11). **e** Percentage of CD3^+^CD4^+^IFN-γ^+^ cells (OA *n* = 12; RA *n* = 11). Differences between groups were calculated using Mann-Whitney *U* test. BM bone marrow, BMMC bone marrow mononuclear cells, FSC forward scatter, IFN interferon, IL interleukin, OA osteoarthritis, RA rheumatoid arthritis, SSC side scatter

The percentage of CD3^+^CD4^+^IL-17^+^ cells was significantly higher in BM of RA patients in comparison to OA patients (1.0% vs 0.6%, *p* < 0.001) (Fig. 2b). Similarly, the percentage of CD3^+^CD4^+^IL-17^+^IFN-γ^+^ cells was higher in BM of RA patients in comparison to BM of OA patients (0.2% vs 0.1%, *p* < 0.03) (Fig. 2c). Both differences were statistically significant. We did not observe significant differences in BM percentage of CD3^+^CD4^+^IL-4^+^ (4.0% vs 3.1%) and CD3^+^CD4^+^IFN-γ^+^ (12.9% vs 7.65%) between RA and OA patients (Fig. 2d, e).

Increased frequency of CD3^+^CD4^+^IL-17^+^ cells in RA BM suggests the recruitment of Th17 cells from the periphery or Th17 cell differentiation/stimulation in situ. Our next experiments were performed in order to test these two possibilities.

### Proinflammatory milieu of RA BM microenvironment

**Fig. 3:**
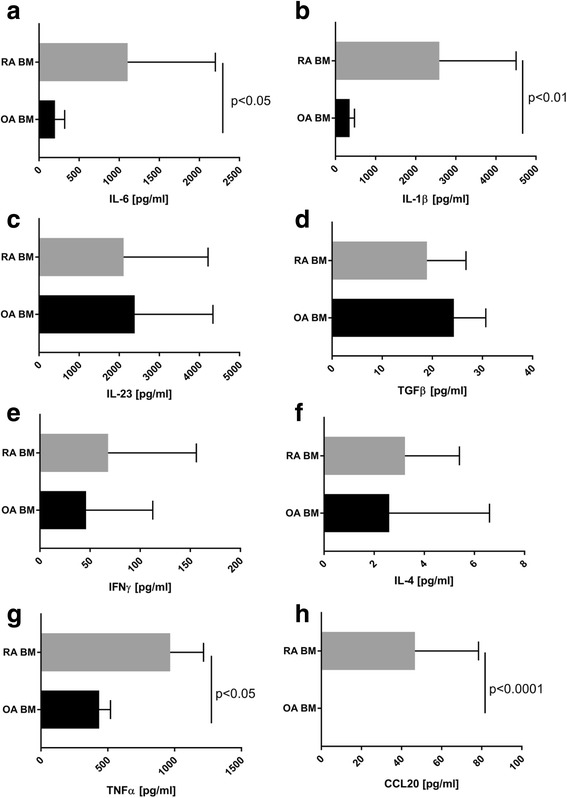
Concentrations of selected cytokines associated with Th17 cell differentiation and activity in BM plasma of RA and OA patients. All cytokine concentrations detected using specific ELISAs. **a–d** Concentration of IL-6, IL-1β, IL-23 and TGF-β (OA *n* = 13–15; RA *n* = 10–14). **e, f** Concentrations of IFN-γ and IL-4 (OA n = 28–29; RA *n* = 27). **g** Concentration of TNF-α (OA *n* = 14; RA *n* = 12). **h** Concentration of CCL20 (OA *n* = 35; RA *n* = 28). Differences between groups were calculated using Mann–Whitney *U* test. BM bone marrow, IFN interferon, IL interleukin, OA osteoarthritis, RA rheumatoid arthritis, TGF transforming growth factor, TNF tumour necrosis factor

In the next step the concentrations of cytokines involved in human Th17 cell differentiation were investigated in BM patient samples. We found an increased concentration of IL-6 (1105 pg/ml vs 198 pg/ml, *p* < 0.05) and IL-1β (2589 pg/ml vs 354 pg/ml, *p* < 0.01) in RA BM plasma in comparison to OA BM plasma (Fig. 3a, b). However, the differences in IL-23 (981 pg/ml vs 2034 pg/ml) and TGF-β (18.9 pg/ml vs 24.3 pg/ml) concentrations in both groups of patients were not statistically significant (Fig. 3c, d). Concentrations of Th1-related cytokine IFN-γ (46 pg/ml vs 68 pg/ml) and Th2-related IL-4 (2.6 pg/ml vs 3.2 pg/ml) were similar in both groups of patients (Fig. 3e, f). In addition, we found an increased concentration of TNF-α, an important player in the proinflammatory cytokine network, in RA BM plasma in comparison to OA BM plasma (966.7 pg/ml vs 435.5 pg/ml, *p* < 0.05) (Fig. 3g). An important chemokine that attracts Th17 cells by binding to receptor CCR6 is CCL20. CCR6 is also a known marker of Th17 cells [20]. Interestingly, we found increased concentration of CCL20 chemokine in RA BM (median value 46.7 pg/ml) in comparison to OA BM (median value 0 pg/ml) (Fig. 3h), which could be responsible for the increased number of Th17 cells in RA BM.

### IL-15 increases IL-17 secretion by RA BMMC

Our previous data indicate that IL-15 concentration is elevated in RA BM [2]. It was also shown that IL-15 levels correlate with IL-17 levels in sera of RA patients [13]. Our present data show that IL-15 concentration correlated with IL-17AF concentration in RA BM but did not correlate with IL-17FF concentration (Table 2).

**Table 2:** Correlation coefficients (*r*) and significance values (*p*) between levels of IL-17AF, IL-17FF and IL-15 in BM plasma of RA patients (*n* = 28)

		*r*	*p*
RA BM IL-17AF	RA BM IL-15	0.38	<0.05
RA BM IL-17FF	RA BM IL-15	0.034	ns

*BM* bone marrow, *IL* interleukin, *ns* not significant, *RA* rheumatoid arthritis

**Fig. 4:**
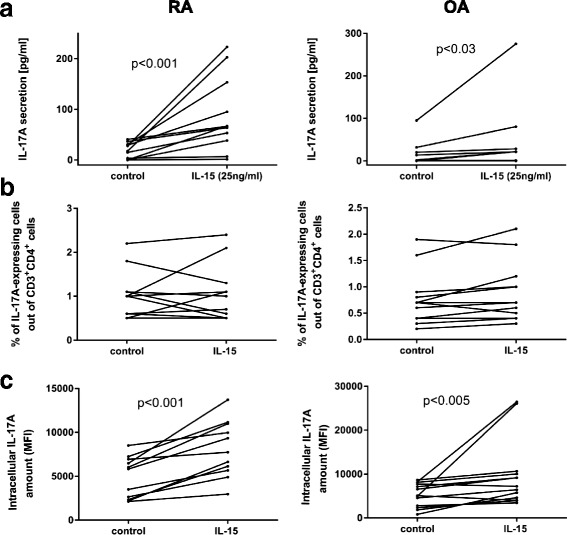
Impact of IL-15 stimulation on IL-17 production and on Th17 differentiation. BMMC from OA (right panel) and RA (left panel) patients cultured for 72 hours without (control) or with addition of IL-15 (25 ng/ml). At the end of cell culture, supernatants were collected for ELISA (**a**) and cells were designated for FACS analysis (**b, c**). **a** Concentration of secreted IL-17A in cell culture supernatants (RA *n* = 11; OA *n* = 11). **b** Percentage of IL-17A-expressing CD3^+^CD4^+^ cells out of total CD3^+^CD4^+^ cells (RA *n* = 11; OA *n* = 13).**c** Intracellular amount of IL-17A (reflected by MFI) in CD3^+^CD4^+^ cells (RA *n* = 11; OA *n* = 13). IL interleukin, MFI mean fluorescence intensity, OA osteoarthritis, RA rheumatoid arthritis

In the next step we investigated the impact of IL-15 stimulation on IL-17 production by BMMC. Although in both groups of patients we observed a statistically significant increase in IL-17A secretion after stimulation with IL-15 (Fig. 4a), such an effect was more profound in BMMC derived from the RA patient group.

### IL-15 does not influence Th17 differentiation

The next experiments were designed to clarify whether IL-15 stimulation influences Th17 cell differentiation. BMMC derived from RA and OA were stimulated for 72 hours with IL-15 and at the end of culture the percentage of IL-17A-positive cells was measured by flow cytometry. In RA patients as well as in OA patients, stimulation with IL-15 did not increase the percentage of IL-17A-producing cells in the culture, suggesting the lack of IL-15 impact on Th17 differentiation (Fig. 4b). However, in both groups of patients an increased intracellular production of IL-17A (mean fluorescence intensity value (MFI)) in CD3^+^CD4^+^ cells was noted upon IL-15 stimulation (Fig. 4c). These results suggest that IL-15 stimulation enhances IL-17A production in existing/already differentiated Th17 cells.

### IL-15 receptor complex is present on CD3^+^CD4^+^ cells isolated from bone marrow

**Fig. 5:**
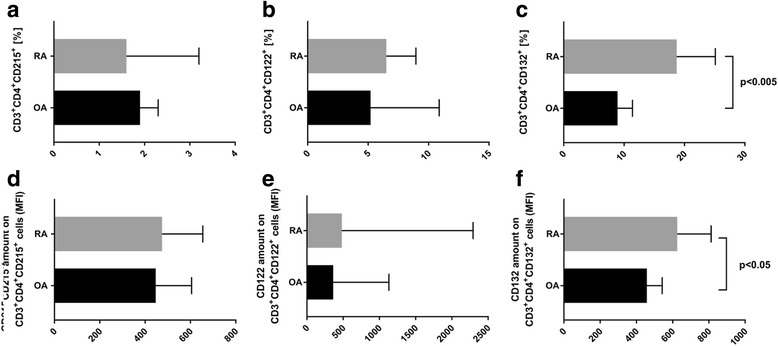
IL-15 receptor complex is present on CD3^+^CD4^+^ cells isolated from RA (*n* = 10) and OA (*n* = 10) BM. Percentage of BM CD3^+^CD4^+^ cells expressing **a** CD215, **b** CD122 and **c** CD132 from RA (*n* = 10) and OA (*n* = 10) patients. MFI value of **d** CD215, **e** CD122 and **f** CD132 on BM CD3^+^CD4^+^ cells from RA (*n* = 10) and OA (*n* = 10) patients. MFI mean fluorescence intensity, OA osteoarthritis, RA rheumatoid arthritis

The heterotrimeric IL-15 receptor complex consists of a unique IL-15Rα subunit (CD215), IL-2/IL-15Rβ (CD122) and the common gamma-chain/IL-15Rγ subunit (CD132). IL-15 binds with high affinity to IL-15Rα (Kd = 10–11 M). IL-15/IL-15Rα then associates with a complex composed of the IL-2/IL-15Rβ and common gamma-chain/IL-15Rγ subunits, expressed either on the same cell (cis presentation) or on a different cell (trans presentation). In some experimental models, IL-15 bound to the IL-15Rα acts much more efficiently than soluble IL-15. We found that the percentage of CD3^+^CD4^+^CD215^+^ cells was similar in BM of RA patients in comparison to OA patients (1.6% vs 1.9%, ns) (Fig. 5a). Also, the percentage of CD3^+^CD4^+^CD122^+^ cells was similar in BM of RA and OA patients (6.5% vs 5.2%, ns) (Fig. 5b). In addition, we did not observe any differences in MFI value of CD215 and CD122 on CD3^+^CD4^+^ cells in both studied patients groups (Fig. 5d, e). However, we noted a significantly higher percentage of CD3^+^CD4^+^CD132^+^ cells in RA BM in comparison to OA BM (18.7% vs 8.9%, *p* < 0.005) (Fig. 5c). Furthermore, MFI value of CD132 on CD3^+^CD4^+^CD132^+^ cells obtained from RA BM was found to be higher in comparison to the same type of cells obtained from OA BM (625 vs 458, *p* < 0.05) (Fig. 5f). Therefore, increased expression of CD132 my be responsible for stronger response of RA BM CD3^+^CD4^+^ cells for IL-15 stimulation.

## Discussion

In the recent years, there has been growing evidence supporting implication of the BM compartment in initiation and perpetuation of the inflammatory processes in RA. Elegant studies confirmed that BM oedema, which is often present in RA patients, reflects true BM inflammation [21]. Moreover, numerous studies have reported that BM oedema represents an independent predictor of RA development and radiographic progression in patients with undifferentiated arthritis [22–26]. Such ongoing inflammatory processes in BM are also reflected by the activation of B cells and T cells. As we reported previously, ligation of Toll-like receptors (TLR) triggered BM-derived B-cell activation [3], and the number of recently activated T cells was significantly elevated in RA BM [2].

Overproduction of several proinflammatory cytokines, including TNF-α, IL-1β, IL-6, IL-15 and relatively recently added IL-17, contribute to pathological processes in RA [6, 27]. Tissue-specific IL-17 exacerbates tissue damage and disease chronicity. It was shown that expression of IL-17 mRNA and protein are higher in RA joints compared to healthy controls [28]. However, there are no data regarding BM production of IL-17 in the course of RA. In the present study we investigated the Th17 compartment in RA BM. In the first sets of experiments the concentrations of IL-17 dimers in BM were measured. We found a significantly higher concentration of IL-17AF in BM of patients with RA than those with OA (Fig. 1). Moreover, in both patient groups, IL-17AF levels in BM plasma were higher than in peripheral blood plasma. There were no differences in BM IL-17FF levels in the OA and RA patient groups. Nonetheless, the IL-17FF concentration in BM plasma was also increased when compared to blood plasma (in both groups of patients). Thus, our results show that BM represents an important source of both IL-17AF and IL-17FF and that overproduction of IL-17AF seems to be characteristic for RA BM.

IL-17 is an important player in the proinflammatory cytokine network. This cytokine may induce production of various proteins in tissues, but when acting alone its effects are not highly pronounced. In contrast, its interaction with other cytokines such as TNF-α, IL-1β, IFN-γ or IL-22 leads to synergistically increased production of IL-6 and IL-8 [29–31]. IL-17 also contributes to increased osteoclastogenesis: it can directly induce differentiation of osteoclasts from monocytes and is able to up-regulate RANKL synthesis by RA fibroblast-like synoviocytes [32]. As we have reported before [33], the RANKL concentration was elevated in RA BM plasma in comparison to OA BM plasma. Thus, the increased production of IL-17A in RA BM may be associated with bone and cartilage destruction observed in the course of RA.

The optimal conditions for Th17 differentiation are still a highly debated issue. There is general agreement that IL-6 and IL-23 participate in the differentiation and survival of murine Th17 [34]. However, even in the murine model, the role of TGF-β remains controversial. For example, there is a study showing that murine, highly pathogenic Th17 lymphocytes differentiate in the absence of TGF-β [35].

Several cytokines, including IL-1β, IL-6, IL-23 and TGF-β, were demonstrated to participate in the differentiation and survival of human Th17 cells. However, it has also been shown that only IL-6 and IL-1β, but not TGF-β, are essential for Th17 differentiation [36]. On the other hand, in some studies the presence of TGF-β is necessary for RORc expression in human CD4 cells [37].

We compared concentrations of IL-1β, IL-6, IL-23 and TGF-β in BM samples obtained from patients with RA and OA in order to investigate whether RA BM creates suitable conditions for Th17 lymphocyte differentiation/survival. We found significantly increased levels of IL-1β and IL-6 in RA BM (Fig. 3a, b). IL-23 and TGF-β were also present in RA and OA BM, although at the comparable levels in both patient groups (Fig. 3c, d). In addition, we observed an increased percentage of Th17 cells in the BM of RA patients compared to the BM of OA patients (Fig. 2), indicating that all necessary cytokines described in the literature required for human Th17 cell differentiation are present in BM in sufficient concentrations to trigger differentiation of Th17 cells. It is likely that levels of TGF-β and IL-23, although not different between RA and OA, are sufficient for supporting Th17 cell differentiation while highly elevated levels of IL-1β and IL-6 may be responsible for the higher number of Th17 cells in RA BM. There are observations that Th1-related and Th2-related cytokines (IFN-γ, IL-4) suppress differentiation of Th17 cells [11, 12]. However, in our experiments we did not observe increased levels of these cytokines in RA BM in comparison to OA BM (Fig. 3e, f).

The role of IL-15 in Th17 cell differentiation is not clearly defined. The IL-15 concentration is known to correlate with the concentration of IL-17 in synovial fluid from RA patients [13]. In addition, the contribution of IL-15 to the increased level of IL-17 in the course of collagen-induced arthritis has been also reported [38]. Our research demonstrated increased production of IL-17 in RA BMMC after IL-15 stimulation (Fig. 4a). Interestingly, flow cytometry experiments showed that although IL-15 did not affect the percentage of BM cells producing IL-17 in vitro, it increased the amount of intracellular IL-17 (referred as MFI, Fig. 4). Additionally, we found correlation between concentrations of IL-17AF and IL-15 in RA BM plasma. These results suggest that IL-15 may contribute to the phenotype stabilization and survival of Th17 cells as well as the increased production of IL-17. It is noteworthy that such an effect is not characteristic only for RA, but also for OA. However, BMMC obtained from RA patients reacted to IL-15 slightly better, indicating the increased sensitivity of T cells from RA BM to IL-15. Our analysis showed the presence of IL-15 receptor complex on CD3^+^CD4^+^ cells isolated from BM. Interestingly, the higher percentage of CD3^+^CD4^+^ cells bearing CD132 present in RA BM than in OA BM may explain why CD3^+^CD4^+^ cells from RA patients react stronger to IL-15 stimulation. It should, however, be noted that the stimulatory effect of IL-15 on IL-17 production in BMMC culture may result from direct stimulation of Th17 cells or other cells capable of producing cytokines involved in Th17 cell differentiation [39].

Another explanation for increased IL-17AF concentration in BM of RA patients could be the increased migration of Th17 cells into the BM. We reported previously the increased levels of some chemokines in BM of patients with RA that indicate the possibility of impaired cell migration in the course of the disease [4]. However, previously we did not evaluate the concentration of CCL20, the key chemokine regulating Th17 migration. CCL20 recruits Th17 cells and dendritic cells to the inflammatory site [20]. CCL20 is typically expressed at a low basal level, but can be strongly induced by proinflammatory cytokine TNF-α [40]. In the present study we found elevated concentrations of CCL20 as well as TNF-α in RA BM plasma. Highly elevated CCL20 in BM of RA patients may participate in the observed increased percentage of Th17 cells as well as the formation of germinal centres in this tissue as we reported before [2].

## Conclusions

Our results indicate that in the course of RA the BM microenvironment can promote the development of Th17 responses and overproduction of IL-17AF. Our observations support the notion that BM actively participates in pathogenesis of RA.

## Abbreviations

7-AAD: 7-Aminoactinomycin D
Ab: Antibody
APC: Allophycocyanin
APC-Cy7: Allophycocyanin tandem conjugate with cyanine
BM: Bone marrow
BMMC: Bone marrow mononuclear cells
ELISA: Enzyme-linked immunosorbent assay
FITC: Fluorescein isothiocyanate
IFN-γ: interferon gamma
IL: Interleukin
MFI: Mean fluorescence intensity
NaN_3_: Sodium azide
OA: Osteoarthritis
PE: Phycoerythrin
PE-Cy7: Phycoerythrin tandem conjugate with cyanine
PerCp-Cy5.5: Peridinin-chlorophyll proteins tandem conjugate with cyanine
PMA: Phorbol 12-myristate 13-acetate
RA: Rheumatoid arthritis
TGF-β: Transforming growth factor beta
Th: T-helper
TNF-α: Tumour necrosis factor alpha
